# In Situ Fabrication of Freestanding Single‐Atom‐Thick 2D Metal/Metallene and 2D Metal/ Metallene Oxide Membranes: Recent Developments

**DOI:** 10.1002/advs.202100619

**Published:** 2021-08-30

**Authors:** Huy Q. Ta, Rafael G. Mendes, Yu Liu, Xiaoqin Yang, Jingping Luo, Alicja Bachmatiuk, Thomas Gemming, Mengqi Zeng, Lei Fu, Lijun Liu, Mark H. Rümmeli

**Affiliations:** ^1^ Soochow Institute for Energy and Materials Innovations College of Energy Collaborative Innovation Center of Suzhou Nano Science and Technology Key Laboratory of Advanced Carbon Materials Wearable Energy Technologies of Jiangsu Province Soochow University Suzhou 215006 China; ^2^ Institute for Complex Materials IFW Dresden P.O. Box D‐01171 Dresden Germany; ^3^ School of Energy and Power Engineering Xi'an Jiaotong University No. 28, Xianning West Road Xi'an Shaanxi 710049 China; ^4^ Material Science & Engineering Center Łukasiewicz Research Network – PORT Polish Center for Technology Development Ul. Stabłowicka 147 Wrocław 54‐066 Poland; ^5^ College of Chemistry and Molecular Science Wuhan University Wuhan 430072 China; ^6^ Centre of Polymer and Carbon Materials Polish Academy of Sciences M. Curie‐Sklodowskiej 34 Zabrze 41‐819 Poland; ^7^ Center for Energy and Environmental Technologies VSB‐Technical University of Ostrava 17. Listopadu 15 Ostrava 708 33 Czech Republic

**Keywords:** 2D metals/metallenes, freestanding single‐atom‐thick membrane, in situ TEM, single‐element 2D materials

## Abstract

In recent years, two‐dimensional (2D) materials have attracted a lot of research interest as they exhibit several fascinating properties. However, outside of 2D materials derived from van der Waals layered bulk materials only a few other such materials are realized, and it remains difficult to confirm their 2D freestanding structure. Despite that, many metals are predicted to exist as 2D systems. In this review, the authors summarize the recent progress made in the synthesis and characterization of these 2D metals, so called metallenes, and their oxide forms, metallene oxides as free standing 2D structures formed in situ through the use of transmission electron microscopy (TEM) and scanning TEM (STEM) to synthesize these materials. Two primary approaches for forming freestanding monoatomic metallic membranes are identified. In the first, graphene pores as a means to suspend the metallene or metallene oxide and in the second, electron‐beam sputtering for the selective etching of metal alloys or thick complex initial materials is employed to obtain freestanding single‐atom‐thick 2D metal. The data show a growing number of 2D metals/metallenes and 2D metal/ metallene oxides having been confirmed and point to a bright future for further discoveries of these 2D materials.

## Graphical abstract

In this review, the authors summarize the recent progress made in the synthesis and characterization of these 2D metals, so called 2D metals/metallenes, and their oxide forms, 2D metals/metallene oxides as free standing 2D structures formed in situ through the use of TEM and STEM to synthesize these materials.

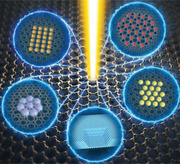

## 1 Introduction

### 1.1 New 2D Materials and Their Freestanding Structure

2D materials can exhibit exceptional surface‐related properties because their atomic‐scale thickness leads to surface and quantum confinement effects, such as the quantum Hall effect,^[^ ^1^, ^2^ ^]^ as well as very high electron mobility^[^ ^3^, ^4^ ^]^ and thermal conductivity.^[^ ^5^ ^]^ Hence, they have been studied intensively in the last decade. 2D materials such as graphene, *h*‐BN, and transition metal dichalcogenides (TMD), which are used widely, are known to exhibit strong intralayer covalent bonding and weak interlayer van der Waals bonding. Thus, they can be exfoliated with ease to form atom‐thick layers. On the other hand, metals with metallic bonding (isotropic) preferentially exhibit 3D close‐packed structures, which presumably cannot be exfoliated to form 2D structures. Therefore, currently, research on 2D materials is mostly limited to van der Waals structures.

Research on 2D metals was initiated with reports of the existence of single^[^ ^6^ ^]^ and few‐layer materials.^[^ ^7^, ^8^ ^]^ For instance, membranes of potassium on graphene, single‐atom‐thick layers of Pb and In on Si(111),^[^ ^9^ ^]^ Hf layers on Ir(111),^[^ ^10^ ^]^ Rh nanosheets on poly(vinylpyrrolidone),^[^ ^11^ ^]^ and Ga layers on different substrates have been reported.^[^ ^12^ ^]^ Hence, the term “metallene” is being used to refer to metals, which do not show a layered structure in the bulk form and can exist as a 2D layered structure. An example would be silicene (the 2D form of silicon).^[^ ^13^ ^]^ Similarly, tin can yield stanene,^[^ ^14^, ^15^ ^]^ while germanene is the 2D form of germanium.^[^ ^16^ ^]^ In addition, antimonene^[^ ^17^ ^]^ and plumbene have also been reported.^[^ ^18^ ^]^ As 2D materials, 2D metals/metallenes are assumed to be sufficiently stable in their freestanding form (as is the case for graphene, h‐BN, and TMD, among other materials). However, this has not yet been demonstrated experimentally. The first freestanding iron membrane with single‐atom thickness was successfully fabricated by Rümmeli et al.,^[^ ^19^ ^]^ in 2014, using an electron beam (e‐beam). This confirmed the existence of 2D metals/metallenes as a new class of 2D materials. In keeping with previous approaches, such as the epitaxial growth of a 2D metals/metallene using a small‐lattice‐mismatch substrate, Rümmeli et al. used the e‐beam of a TEM system to drive the formation of Fe surface atoms and nanoparticles (NPs) into Fe 2D membranes suspended in graphene pores. This increased research interest in freestanding 2D metals/metallenes as well as the possibility using e‐beams to form novel 2D materials.

Inspired by the discovery of Rümmeli et al., there have been several theoretical efforts to predict the existence of other freestanding 2D metals/metallenes, such as those of Au,^[^ ^20^ ^]^ Ag,^[^ ^21^ ^]^ and Cu.^[^ ^22^ ^]^ In a systematic investigative study by Nevalaita et al.,^[^ ^23^ ^]^ the 2D structures of 45 different metals were analyzed through density functional theory (DFT) simulations. The calculations showed that most of the 2D metals are more stable in the hexagonal‐ and honeycomb‐structured forms than in the square‐lattice‐structured form. The cohesive energy plays an important role in the formation of 2D metals. A stable 2D metal must exhibit a balance between the 2D cohesion, which must be sufficiently high, and the 3D bulk cohesion, which must be sufficiently low. The 2D structure will be unstable if the 2D cohesion is too low, while a 3D structure is formed if the 3D bulk cohesion is too high. Hence, both theoretical and early experimental studies suggest the existence of a new class of 2D elemental metals in the form of freestanding structures.

### 1.2 Electron Beam–Specimen Interactions

As discussed in the previous section, TEM is not merely a simple tool for characterizing 2D materials. During observations, its e‐beam also interacts with the test specimen. For instance, in the case of Fe NPs, it can drive the NPs to form a monoatomic Fe membrane. This is a typical example highlighting the power and versatility of advanced high‐resolution TEM (HRTEM) systems in materials science, physics, and chemistry. The energetic electrons can not only yield morphologic, compositional, and crystallographic information regarding the test specimen but can also drive in‐situ reactions with the specimen (i.e., e‐beam–specimen interactions) at the atomic level.^[^ ^19^, ^24^, ^25^, ^26^, ^27^, ^28^, ^29^, ^30^ ^]^ Various types of interactions can occur during TEM^[^ ^31^ ^]^ when the energetic electrons penetrate the test specimen. The incident electrons lose their energy (or experience a change in their momentum), and this energy is transferred to the specimen atoms, resulting either in the heating of the specimen or the breakage and rearrangement of the chemical bonds between its atoms. On the other hand, a few electrons can travel through the specimen without any (or very little) loss in energy. These two events can be categorized as corresponding to two different types of electron interactions, namely, inelastic and elastic interactions.^[^ ^31^ ^]^

In the case of inelastic interactions, the incident electrons transfer a certain amount of energy to the atomic electrons in the specimen. This transferred energy can generate different signals, such as Auger or secondary electrons, X‐rays, plasmons, and phonons.^[^ ^31^, ^32^ ^]^ The signals produced by inelastic interactions are mainly employed in analytical electron microscopy methods, such as electron energy loss spectroscopy (EELS) and energy‐dispersion X‐ray spectroscopy. In addition, such inelastic interactions may also cause various effects, such as specimen heating, ionization‐induced damage (radiolysis), and hydrocarbon contamination. In the case of elastic interactions, the electrons do not transfer any energy (or only a negligible amount of energy) to the specimen. Such electrons are used in the case of TEM and electron diffraction analysis methods. In addition, these elastically scattering electrons may induce various phenomena such as electrostatic charging, atomic displacement, and e‐beam sputtering.^[^ ^31^, ^32^ ^]^ These e‐beam‐induced phenomena can be exploited for the in‐situ TEM analysis of 2D materials.

### 1.3 In Situ (S)TEM Analysis of New 2D Materials

**Figure 1:**
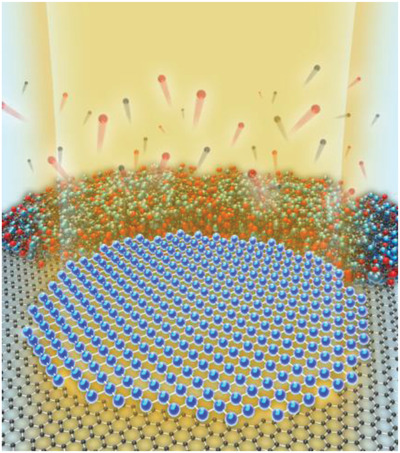
Elastic and inelastic e‐beam–specimen interactions during in situ (S)TEM induce formation of freestanding 2D metal/metal oxide membrane. Electron beam (semi‐transparent orange shading) interacts with specimen atoms (red, blue, black balls) to form a 2D free‐standing membrane (blue stick and ball lattice) from 3D NPs (clusters of red, blue, black atoms) in the graphene hole (black stick and ball honeycomb lattice indicating graphene). Reproduced with permission.^[^ ^36^ ^]^ Copyright 2020, American Chemical Society.

High‐resolution (scanning) TEM (HR(S)TEM) with advanced upgraded spherical aberration (Cs) correction and reduced chromatic aberration (particularly for low acceleration voltages) has emerged as an ideal technique for studying the lattice structure of advanced materials and, in particular, 2D materials, which become reactive under an e‐beam. The subatomic spatial resolution of advanced HR(S)TEM allows one to visualize the atomic structure and bonding configuration of materials and track the movement of their atoms with high temporal resolution. Furthermore, the elastic and inelastic interactions of the electrons with the specimen, which may induce radiolysis, hydrocarbon contamination, atomic displacement or sputtering, bonding rearrangement, and crystallization, can be usefully exploited for the in‐situ (S)TEM e‐beam‐driven chemical reaction studies of (e‐beam‐reactive) 2D materials,^[^ ^29^, ^30^, ^33^, ^34^, ^35^ ^]^ such as those related to their growth, etching, and reduction/oxidation. During such in‐situ (S)TEM studies, the e‐beam plays the dual role of inducing the reactions while simultaneously recording them. This is extremely important because the synthesis of 2D materials requires a comprehensive understanding of their characteristics, and in‐situ observations of their growth process at the atomic level can provide this information. In other words, (S)TEM can elucidate the growth mechanism of 2D materials. In addition, while in the case of other methods for synthesizing 2D metals/metal oxides, it may be challenging to demonstrate the freestanding structure of the synthesized material; this difficulty can be overcome using in‐situ (S)TEM (**Figure** **1**) and graphene pores as templates^[^ ^19^, ^24^, ^36^, ^37^, ^38^, ^39^, ^40^ ^]^ or e‐beam‐based thinning methods.^[^ ^41^, ^42^, ^43^, ^44^ ^]^ In this review, we summarize the recent progress made in the in situ e‐beam‐driven synthesis of freestanding 2D metal/metal oxide membranes using(S)TEM and highlight the importance of in situ (S)TEM in the development of freestanding 2D materials.

## 2 Graphene Holes: Suspension System for In Situ Fabrication of Freestanding Single‐Atom‐Thick 2D Metals/Metallenes and 2D Metals/Metallenes Oxides

In addition to exhibiting several desirable properties, graphene shows preferential in‐plane adsorption^[^ ^45^, ^46^ ^]^ at its edges instead of on‐top adsorption;^[^ ^47^ ^]^ this results in exothermic interactions (in the suitable direction) between the atoms of a metal layer formed on it and the pore edges of graphene, which, in turn, help stabilize the 2D membranes formed within the graphene pores.^[^ ^48^ ^]^ Owing to this phenomenon of stabilization of 2D metal/metal oxide membranes, the use of graphene pores as growth templates is an extremely promising approach for the growth of freestanding 2D metal/metal oxide membranes.

### 2.1 Electron‐Beam‐Driven Formation of Suspended 2D Metals/Metallene Membranes

**Figure 2:**
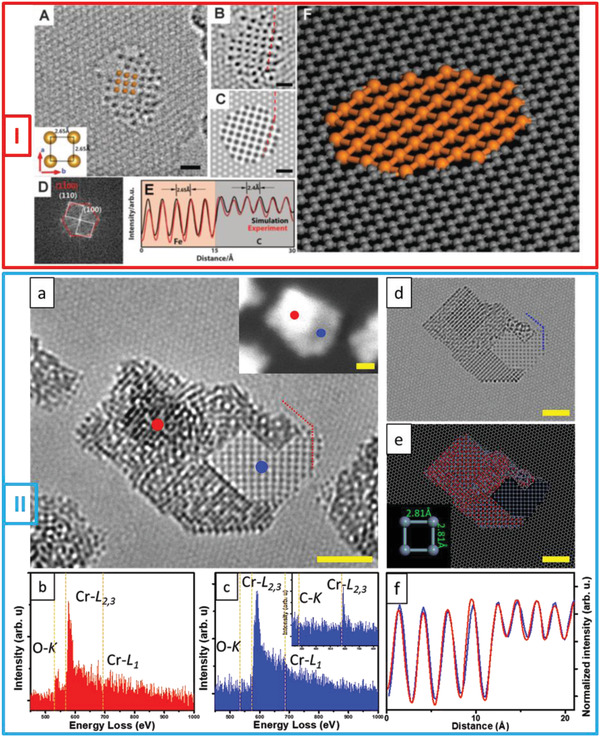
E‐beam‐driven formation of 2D metals/metallene membrane. Panel I: Freestanding monoatomic 2D Fe layer. A) HRTEM image of freestanding single‐atom‐thick 2D Fe membrane with square unit cell in graphene pore. Interatomic spacing of square unit cell is highlighted in inset. B) Filtered HRTEM image from (A). C) Simulation image of single‐atom‐thick 2D membrane Fe. D) Fast Fourier transform (FFT) of image of structure in (A) showing relationship between lattices of graphene substrate (red) and monoatomic Fe layer (white). E). Matching intensity profiles (i.e., normalized intensity profiles from simulation image (black line) and experimental image (red line) corresponding to marked profiles represented by red dashed lines in (B) and (C)) confirming that Fe membrane is single‐atom thick. F) Stick‐and‐ball model showing atomic structure of monoatomic 2D Fe membrane suspended within graphene pore. All scale bars: 0.6 nm. Reproduced with permission.^[^ ^19^ ^]^ Copyright 2014, American Association for the Advancement of Science. Panel II: Characterization of monatomic 2D Cr membrane between graphene and CrO NPs. a) HRTEM micrograph showing freestanding single‐layer 2D Cr membrane with square lattice between CrO NP and graphene. Inset STEM image obtained at same position as HRTEM image; red and blue dots highlight positions where local EELS profiles were acquired. b,c) Local EELS profiles of CrO NP (red) and 2D Cr membrane (blue) measured at areas represented by red and blue dots, respectively, in (a). Inset EELS profiles, which does not contain C signal, corresponds to membrane, indicating 2D membrane is freestanding. d,e) Simulation image and stick‐and‐ball model for structures in TEM image in panel (a) for comparison. Interatomic spacing of square unit cell is highlighted in inset. f). Matching normalized intensity profiles (normalized with respect to graphene) from TEM image (red curve) and simulation image (blue curve) corresponding to red and blue dashed lines in (a) and (d), respectively). Profiles indicate Cr membrane is one‐atom thick. All scale bars: 2 nm. Reproduced with permission.^[^ ^36^ ^]^ Copyright 2020, American Chemical Society.

As discussed previously, a single‐atom‐thick 2D Fe membrane was the first freestanding 2D metals/metallene grown in graphene pores using an e‐beam^[^ ^19^ ^]^ (**Figure** **2** (Panel I)). For this, an 80‐kV e‐beam of a low‐voltage spherical‐aberration‐corrected TEM (LVACTEM) system was used to drive the formation of Fe nanocrystals to produce a single‐atom‐thick 2D crystalline Fe membrane. The pure Fe atoms, clusters (or NPs) residing near graphene holes could be observed readily. These holes were formed using a Cu etchant (FeCl_3_)^[^ ^49^ ^]^ during the transfer process or intentionally using a condensed electron beam. Under e‐beam irradiation, the Fe nanocrystals located near the graphene pores started to collapse into a disordered structure. The Fe atoms that were freely mobile on the graphene substrate under the e‐beam were attracted by the graphene hole edges and moved toward the holes, recrystallizing into a square lattice and forming a monolayer 2D Fe membrane suspended within the graphene pores. The results of DFT calculations were consistent with this finding, in that they also indicated that the formation of a single‐layer 2D square lattice Fe membrane is energetically favored instead of the other possible 2D structures, such as tetragonal and hexagonal ones. The experimentally measured lattice constant for this membrane (2.65 ± 0.05 Å) was larger than that of the (200) face‐centered cubic phase or the (110) body‐centered cubic (bcc) phase of 3D bulk Fe. This increase in the lattice constant (near the membrane center) is attributable to the lattice alignment and mismatch between the graphene substrate and the 2D Fe monolayer, which results in a strain. In addition, the calculations also showed that the magnetic moment of single‐atom‐thick Fe membranes (3.08 μB) is significantly higher than that for bulk bcc Fe (2.1 μB). The largest stable membrane as per the experimental data and DFT calculation results was 10–12 atoms in width.

In another experimental study, Ta et al. reported the in‐situ synthesis of a freestanding single‐atom‐thick 2D metals/metallene using an e‐beam.^[^ ^36^ ^]^ A single‐layer 2D antiferromagnetic Cr membrane was formed (Figure 2 (Panel II)) using the same graphene‐pores‐as‐template approach. The Cr source was deposited on graphene through sublimation. The subsequent decomposition of Cr acetyl acetonate (acac) occurred at an elevated temperature, resulting in C, Cr, and O species, which were deposited and formed a thin composite film on the graphene surface. The in‐situ formation of the freestanding 2D Cr membrane using an e‐beam was a two‐step process: i) the deposited Cr‐O‐C amorphous composite film was subjected to electron irradiation to preferentially sputter C, producing chromium oxide (Cr*_x_* O*_y_*) crystals. ii) O was then preferentially sputtered from the oxide nanocrystals under continued e‐beam irradiation, leading to the formation of a freestanding 2D single‐atom‐thick Cr membrane, which was suspended within the graphene pores. The local EELS profiles were measured to confirm that the membrane comprised pure Cr. In addition, the EELS spectra of the Cr‐L_2,3_ edges from different Cr based forms (amorphous Cr/O/C composite, Cr*_x_* O*_y_* NPs, bulk Cr and 2D Cr membrane) were studied further. The Cr‐L_3_/L_2_ white‐line intensity ratio from the 2D Cr membrane (<1) was found to be significantly smaller than that compared to other structures reflecting the different bonding configuration of the 2D single atom thick Cr membrane as compared to the other Cr forms. DFT calculations indicated that the ground state of the 2D Cr membrane was antiferromagnetic, making it suitable for spintronic applications and further highlighting the existence of a new class of 2D metals/metallenes.

### 2.2 Electron‐Beam‐Driven Formation of 2D Metals/Metallenes as Nanoribbons and Planar Clusters

**Figure 3:**
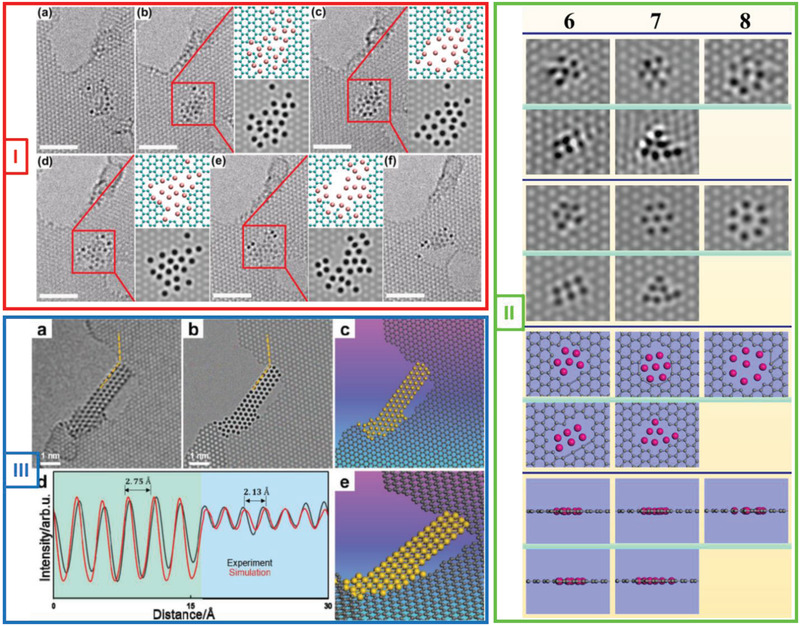
E‐beam‐driven formation of 2D metals: Nanoribbons/clusters. Panel I: E‐beam‐driven transformation of Pt cluster embedded in graphene into planar structure in graphene pore. Stick‐and‐ball model and corresponding simulation image of highlighted region are shown in insets. Scale bars: 2 nm. Reproduced with permission.^[^ ^37^ ^]^ Copyright 2013, The American Society of Mechanical Engineers. Panel II: Example of in‐plane single‐atom‐thick 2D Sn patches embedded within graphene pores under e‐beam irradiation. From top to bottom: HRTEM images, simulation images, top‐view stick‐and‐ball models, and side‐view stick‐and‐ball models for different types of clusters. Reproduced with permission.^[^ ^38^ ^]^ Copyright 2020, Springer Nature. Panel III: Formation of freestanding 2D Au nanoribbon within graphene pore under e‐beam irradiation. a,b) HRTEM image and simulation image, respectively, showing single‐atom‐thick 2D Au nanoribbon suspended between two graphene edges. c,e) Top view and 3D view of stick‐and‐ball model of freestanding 2D Au nanoribbon. d) Matching intensity profiles (normalized intensity profiles from simulation image (red line) and experimental image (black line)) corresponding to profiles marked by dashed orange lines in (a, b), indicating that Au nanoribbon is single‐atom thick. Reproduced with permission.^[^ ^39^ ^]^ Copyright 2020, Wiley‐VCH.

2D metals do not always form a crystalline freestanding membrane under e‐beam irradiation. Freestanding 2D metal clusters have also been observed. For example, 2D metal nanoclusters suspended within graphene holes were first reported by Dong et al.^[^ ^37^ ^]^ They observed small Pt clusters embedded within graphene pores. Under e‐beam irradiation, these clusters gradually transformed into a planar structure, as shown in **Figure** **3** (Panel I). Owing to edge reconstruction, the configuration of the Pt clusters changed constantly during the e‐beam irradiation process. The catalytic effect of the low‐coordinated Pt clusters aided this phenomenon. Under prolonged irradiation, a Pt–graphene composite structure was formed by the intermixing of the carbon and Pt atoms (Panel I(c)). Since the carbon atoms in the Pt clusters were sputtered, the resulting pure Pt clusters changed into a planar structure, with the holes increasing in size continuously (Panel I(e)). Under e‐beam irradiation, the carbon atoms were constantly sputtered from the graphene lattice, leading to the expansion of the graphene pores (Panel I(f)). The planar Pt clusters finally collapsed into a 3D structure once the hole size was larger than the maximum extendibility of the Pt clusters.

Yang et al.^[^ ^38^ ^]^ also reported observing not a 2D metals/metallene (i.e., stanene) but novel 2D single‐atom‐thick Sn membranes suspended within graphene pores, as shown in Figure 3 (Panel (II)). The Sn atoms were deposited on graphene from Sn acac (this approach was similar to that employed by Ta et al.^[^ ^36^ ^]^) under irradiation from the e‐beam (80 kV) of an LVACTEM system. This resulted in novel single‐atom thick 2D planar clusters/membranes with sizes of 1–8 atoms embedded within the graphene pores. A star‐like or close‐packed structural configuration was observed in the case of the patches of three or more atoms.

The team also employed DFT studies to look at the cluster configurations and energetics. The results deviated from experimental observations for membranes larger than five atoms and this was attributed to the interfacial forces between the graphene pore edges and Sn atoms not having been including in the calculations. However, in initial interfacial study looking at the adsorption energetics of a Sn atom adsorbed on graphene or within a C vacancy was conducted and the study confirmed that pores can stabilize Sn atoms through interactions with the graphene pore edges.

In a further study, freestanding single‐atom‐thick 2D Au nanoribbons, which are another form of freestanding single‐atom‐thick 2D metal structures, were reported by Zhao et al.^[^ ^39^ ^]^ Under e‐beam irradiation, the shape of the underlying graphene pores change, leading to the dynamic transformation of the Au nanoclusters into freestanding 2D membranes, which subsequently turn into freestanding 2D nanoribbon with a ribbon width of 4–7 atoms. The Au ribbon structures match that of the top monolayer of bulk Au (111), viz, a hexagonal close‐packed lattice matching very well with previous theoretical predictions.^[^ ^45^ ^]^ Moreover, the theoretical predictions suggest 2D single‐atom‐thick Au structures are metallic and diamagnetic with metallic bonds holding the gold atoms together in the 2D plane. Experimentally, these freestanding 2D Au nanoribbons showed good stability under irradiation with an 80‐kV e‐beam.

### 2.3 Electron‐Beam‐Driven Formation of Suspended 2D Metals/Metallene Oxides

**Figure 4:**
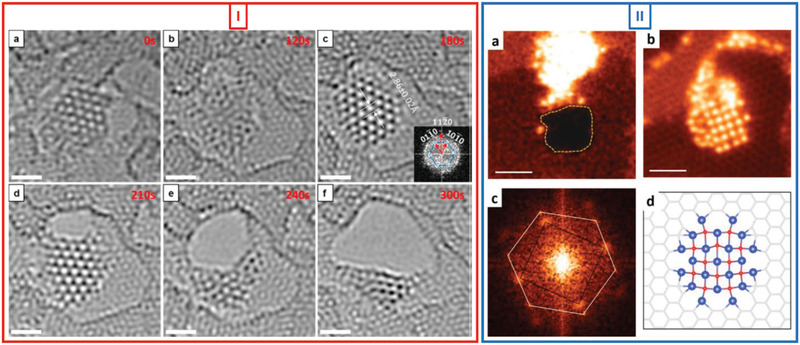
E‐beam‐driven formation of freestanding 2D metal oxides. Panel I: HRTEM images showing in‐situ e‐beam‐driven formation of freestanding 2D g‐ZnO in graphene pore. a) Small ZnO nanocrystal (≈1–2 nm) on graphene substrate. b) ZnO nanocrystal changes into amorphous structure under e‐beam irradiation. c) E‐beam‐driven recrystallization, resulting in single layer of g‐ZnO; inset shows FFT of TEM image of 2D g‐ZnO crystal. d–f) Extended e‐beam irradiation leads to collapse of g‐ZnO structure. All scale bars: 1 nm. Reproduced with permission.^[^ ^24^ ^]^ Copyright 2015, American Chemical Society. Panel II: E‐beam‐driven formation of freestanding monolayer 2D CuO in graphene pore. a) CuO cluster at edge of graphene pore. b) Freestanding monolayer 2D CuO formed in graphene pore. c) FFT of image of 2D CuO membrane and graphene in (b). d) Theoretical stick‐and‐ball model of 2D single‐layer CuO membrane suspended in graphene pore. Scale bars: 1 nm. Reproduced with permission.^[^ ^40^ ^]^ Copyright, 2016 IOP Publishing.

In addition to being suitable for growing freestanding 2D metals/metallenes, e‐beam irradiation over graphene pores as templates can also be used to grow 2D metal oxides. Ta et al.^[^ ^24^ ^]^ reported the e‐beam‐driven in‐situ formation of freestanding graphene‐like ZnO (g‐ZnO) mono‐ and bilayer membranes in graphene pores from ZnO NPs using an LVACTEM system at 80 kV. Theoretical calculations and experimental studies have shown that the bulk structure of ZnO (wurtzite) with a thickness of a few atoms can transform into a supported graphene‐like planar structure.^[^ ^50^, ^51^ ^]^ However, freestanding 2D membranes of g‐ZnO had not been realized until first reported by Ta et al.^[^ ^24^ ^]^ The lattice constant of the transformed g‐ZnO membranes from the membrane center towards the graphene–membrane interface at the outer membrane edges was higher by 1.6% (*a* = 3.303 Å). This increase can be attributed to the lattice mismatch between the graphene substrate and the 2D g‐ZnO membrane. ZnO was obtained by the thermal decomposition of Zn acac under high vacuum, which led to the formation of ZnO (wurtzite) NPs over the graphene substrate. Under e‐beam irradiation, the small ZnO NPs (outer diameter of ≈1–2 nm) formed on the graphene substrate were highly reactive, and their crystal structure changed readily to an amorphous one, in which the Zn and O atoms were highly mobile. Under extended irradiation, the dynamic Zn and O atoms recrystallized, forming a single‐layer graphene‐like structure (**Figure** **4** Panel (I)). The measured d‐spacing was 2.85–2.86 (±0.05) Å and in keeping with the results of previous theoretical and experimental studies.^[^ ^50^, ^51^ ^]^ The freestanding nature of the 2D g‐ZnO membrane in the graphene pores was confirmed by subjecting the TEM images of the membrane to FFT filtering. The g‐ZnO membrane was also relatively stable during the irradiation process, similar to the previously mentioned Fe membrane.

Figure 4 (Panel (II)) shows another example of the formation of a freestanding 2D membrane in graphene pores under e‐beam irradiation using TEM in the scanning mode.^[^ ^40^ ^]^ A single‐atom‐thick copper oxide membrane with a square lattice was formed from a copper oxide nanocrystal under e‐beam irradiation in the STEM mode at 60 kV. Small clusters of copper oxide (<2 nm in diameter) were attached to the step edges of multilayered graphene flakes (Panel II(a)). Driven by a scanning focused e‐beam (60 kV) used in the STEM mode, these clusters moved around the graphene edges, and their morphology changed continuously. At the critical point, they transformed into a crystalline single‐layer structure, with the Cu atoms forming a square sublattice. The experimentally measured value of the lattice constant, d_Cu‐Cu_, was 2.69 ± 0.05 Å and in agreement with the results of the DFT calculations for CuO (d_Cu‐Cu_ of 2.67 Å). The small copper oxide clusters close to the graphene holes also transformed into a monolayer freestanding membrane under low‐dose‐rate e‐beam irradiation. A suspended monolayer with a square Cu sublattice is displayed in the STEM‐annular dark‐field (ADF) image in Panel II(b)), and the FFT of the image is shown in Panel II(c). The experimentally measured d_Cu‐Cu_ value is 2.65 Å, which is also close to the DFT‐calculated value of 2.7 Å for freestanding monolayer CuO in graphene pores (Panel II(d)).

## 3 In‐Situ Electron‐Beam‐Driven Thinning and Restructuring as Means of Forming Suspended 2D Metals/Metallenes

A strong understanding of the underlying theory and precision in the manipulation of materials are essential for achieving the desired control over material properties. The manipulation and engineering of materials at the atomic scale can be achieved through different methods, such as e‐beam irradiation,^[^ ^52^, ^53^ ^]^ ion‐beam irradiation,^[^ ^54^, ^55^, ^56^, ^57^ ^]^ laser irradiation,^[^ ^58^ ^]^ plasma treatment,^[^ ^59^, ^60^ ^]^ scanning electron microscopy (SEM),^[^ ^61^ ^]^ and chemical^[^ ^62^ ^]^ and thermal treatments.^[^ ^63^, ^64^ ^]^ Herein, we focus on approaches that use TEM in the conventional and scanning (STEM) modes to etch or thin 2D materials in a layer‐by‐layer manner to form atomically thin regions as well as novel materials.

The thinning of materials through e‐beam irradiation can occur through elastic and inelastic scattering. These scattering processes are governed by different factors, such as the nature of the material in question and the energy/dose of the incident e‐beam. Furthermore, these processes can give rise to different phenomena, such as charging, atom displacement, sputtering, heating, and radiolysis. These phenomena may induce damage in the irradiated material.^[^ ^65^, ^66^ ^]^ However, e‐beam irradiation, when used with precision in an assisted manner, can be employed advantageously to engineer materials in a controllable manner. For instance, material engineering can be performed with precision using a TEM system. This is because TEM allows for the simultaneous high‐resolution imaging and monitoring of the in situ dynamic transformations that occur during the e‐beam irradiation process. The real‐time monitoring of the material transformations also allows for the irradiation parameters to be changed. Thus, the desired results can be achieved with a high degree of control.

**Figure 5:**
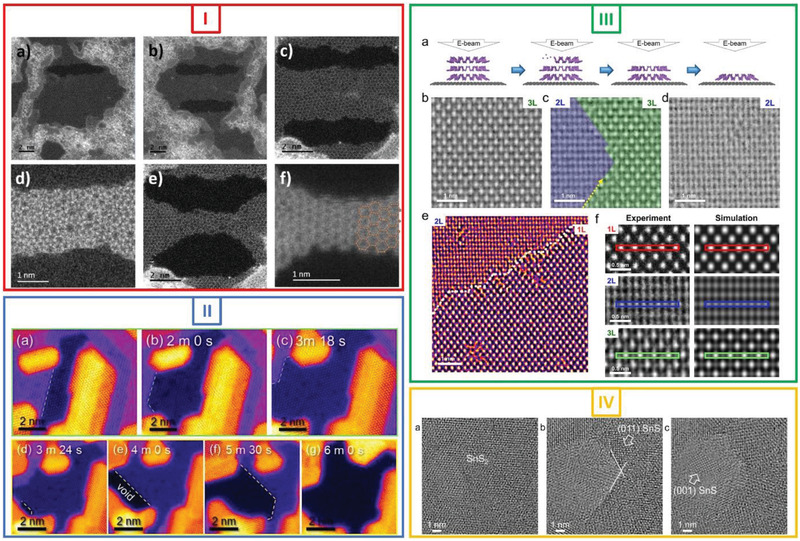
E‐beam thinning of 2D materials. Panel I: Fabrication of a 1 nm wide arm‐chair graphene nanoribbon. a) STEM image of the first mill at 100 kV. b) STEM image after both cuts were made through the graphene. c) Higher magnification image of the formed nanoribbon. d) Further thinning of the central section of the nanoribbon was performed by scanning with the 100 kV beam, one frame of which is shown here. e) STEM image showing the nanoribbon state after milling and thinning. Many defects are present in the thinnest portion of the nanoribbon. The microscope was then switched to 60 kV and the nanoribbon irradiated with the defocused probe during microscope realignment. After alignment the image shown in (f) was acquired. Defects in the nanoribbon healed under the gentler 60 kV irradiation and a pristine arm‐chair nanoribbon was successfully formed. Reproduced with permission.^[^ ^66^ ^]^ Copyright 2020, Elsevier B.V. Panel II: Comparison of the etching speeds of the remaining bilayer and monolayer. a–c) Time series of the STEM images of the expansion of the localized thin area by etching the upper layer. d–g) Time series of the STEM images followed by (a–c), which reveal the layer that had been rapidly etched and soon became a large void. Reproduced with permission.^[^ ^67^ ^]^ Copyright 2020, American Chemical Society. Panel III: Controlled etching of phosphorene to monolayer. a) Schematic illustration of e‐beam‐induced etching process. b−d) Time series of HR‐TEM images showing the thinning from trilayer to bilayer. e) HR‐TEM image showing bilayer and monolayer phosphorene. Color‐code fire of ImageJ program was used. f) Experimentally observed HR‐TEM image and simulated image for monolayer, bilayer, trilayer. Reproduced with permission.^[^ ^68^ ^]^ Copyright 2019, American Chemical Society. Panel IV: Electron‐beam induced thinning of SnS_2_ and transformation of ultrathin SnS_2_ to (001) SnS. a) Initial few‐layer SnS_2_ crystal. b) Simultaneous local thinning and transformation of thicker (001) SnS_2_ to off‐axis (011) SnS (arrow). c) Transformation of ultrathin (001) SnS_2_ to (001) SnS (arrow). Reproduced with permission.^[^ ^69^ ^]^ Copyright 2016, American Chemical Society.

The aim of such manipulations is to ultimately fabricate materials with specific properties that can be used in next‐generation technologies. For example, Dyck et al.^[^ ^66^ ^]^ showed that by varying the electron beam acceleration voltage it is possible to controllably cut and thin graphene monolayers using a focused electron beam. **Figure** **5** Panel I presents their results to obtain graphene nanoribbons using multiple electron beam energies. Tseng et al.^[^ ^67^ ^]^ reported that they were able to thin a few layers of tungsten disulfide (WS_2_) using TEM and STEM. They demonstrated that an e‐beam produced using an acceleration voltage of 200 kV caused the local thinning of WS_2_. Furthermore, the changes in the structure could be monitored, yielding insights into the development of new techniques for the nanoscale modification of 2D WS_2_ as depicted in different snapshots of Figure 5 Panel II during the irradiation of 6 min.

Mono‐ and bilayer phosphorene could be produced through the layer‐by‐layer etching of black phosphorus (BP) using an e‐beam as shown in Figure 5 Panel (III).^[^ ^68^ ^]^ To control the damage caused by the irradiated electrons and consequently control the number of phosphorene layers with precision, the authors used graphene as a protective layer for the multilayered BP. The graphene layer acted as a barrier to prevent radiolysis damage and increase the stability of the BP. The authors also tested different configurations of the graphene/BP heterostructure, namely, graphene on the top of the BP, graphene at the bottom of the BP, and graphene as an encapsulating layer for the BP. The presence of graphene on only one side (top or bottom) of the BP layers allowed for controllable thinning under e‐beam irradiation. On the other hand, in the case of the encapsulated BP configuration, the amorphization of the BP layers was observed. The higher stability of the graphene/BP heterostructure allowed the etching mechanisms in play during the e‐beam irradiation process to be investigated. Thus, it was shown that graphene can be used to aid the e‐beam thinning of 2D materials as well as for detailed investigations of the defects of such materials and their effects on the material properties.

Sutter et al.^[^ ^69^ ^]^ demonstrated that it is possible to controllably remove S and Se atoms from 2D tin dichalcogenides (SnS_2_ and SnSe_2_) using an e‐beam, (Figure 5 Panel IV). The electron irradiation process initially induces the local amorphization of SnS_2_ and SnSe_2_; this is followed by the formation of SnS and SnSe domains. Their results could be reproduced using a heat source, which increased the homogeneity of the formed domains. Moreover, the entire process of the removal of S and Se could be monitored using electron diffraction while the transformations in the material structure were observed for different acceleration voltages (80, 200, and 300 kV).

Chen et al.^[^ ^70^ ^]^ described the etching of MoS_2_ monolayers with zigzag edges. To accomplish this, they annealed the MoS_2_ sample at 800 °C with a blanked electron beam and then tracked the sulfur vacancies caused by electron irradiation with a 60 kV beam. This atom‐by‐atom approach also allowed the formation of MoS_2_ nanoribbons atom‐by‐atom. Tai el al.^[^ ^71^ ^]^ also modified the structure of MoS_2_ layers through the use of an electron beam and heating, leading to the etching and sculpting of multiple MoS_2_ structures in the electron‐irradiated area. The in situ work showed the possibility to etch, fold, and scroll MoS_2_ monolayers. Moreover, their results demonstrated the possibility to create hybrid lateral heterojunctions composed of 1H, 2H, 3R, 3R’, and TZ stacking configurations.

Das et al.^[^ ^72^ ^]^ used aberration‐corrected STEM to etch a MoS_2_/WS_2_ heterostructure in a layer‐by‐layer manner until the formation of nanopores that were arranged in different arrays across the 2D lattice. The thinning process was performed at room temperature using a TEM system at 80 kV. By varying the focus of the e‐beam in the STEM mode, they observed that the MoS_2_ layer was etched first. This was followed by the ablation of the WS_2_ layer, resulting in the formation of pores/defects in the irradiated region. The size of these pores/defects could be controllable to 0.6 nm.

**Figure 6:**
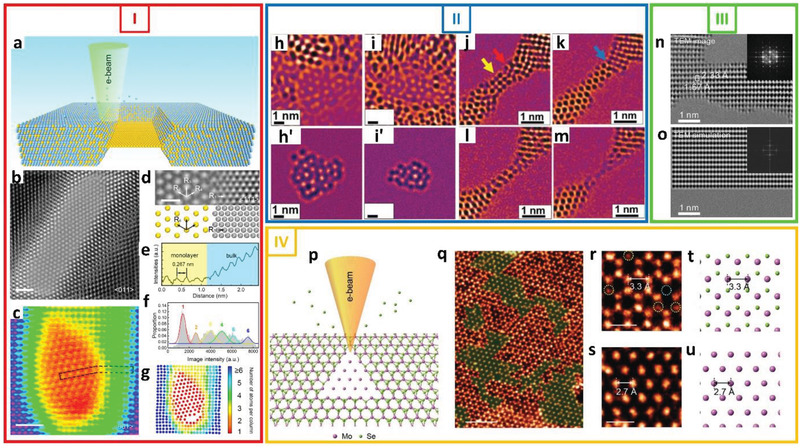
E‐beam thinning of 2D materials. Panel I: E‐beam‐driven‐formation of 2D Au membrane through thinning of Au–Ag alloy. a) Schematic of fabrication of monolayer Au membrane by in‐situ dealloying of Au−Ag alloy. Au and Ag atoms are shown as yellow and blue spheres, respectively. b) HRTEM image of typical monolayer Au membrane with hcp coordinate lattice framed in bulk Au−Ag alloy along ⟨011⟩ zone axis. c) HRSTEM image of monolayer Au membrane framed in bulk Au−Ag along ⟨001⟩ zone axis. d) HRTEM image of monolayer Au membrane and bulk Au−Ag alloy as viewed along ⟨111⟩ zone axis. e) Line profile of HRSTEM image intensity showing monolayer (yellow‐shaded) and bulk (blue‐shaded) regions along black dashed box in (c). f) Histogram of HRSTEM image intensities corresponding to atom columns. Peaks are shown as differently colored Gaussian curves fitted from histogram. g) Top view of atomic map of Au membrane. Atom positions and intensities were measured from image in (c). Scale bars: 1 nm in (b) and (c) and 0.5 nm in (d). Reproduced with permission.^[^ ^41^ ^]^ Copyright 2019, American Chemical Society. Panel II: E‐beam thinning of Mo_2_Ti_2_C_3_ monolayers. h–i) FFT‐filtered high‐resolution TEM images of N‐doped graphene membrane exhibiting structural changes induced by e‐beam irradiation. Defective structure composed of pentagons, hexagons, and heptagons can be seen. h’–i’). Simulation images based on stick‐and‐ball model, confirming presence of N‐doped graphene membrane undergoing structural changes during e‐beam irradiation. j–m) Depict structural changes in Mo nanoribbons upon electron beam irradiation until the formation of a single atom Mo chain. Reproduced with permission.^[^ ^42^ ^]^ Copyright 2020,Wiley‐VCH. Panel III: Characterization of freestanding ultrathin Mo membrane with low‐symmetry rectangular lattice structure. n) TEM image of rectangular lattice structure of ultrathin Mo membrane. Inset shows corresponding Fourier‐transformed image. o) Simulated HRTEM image of two‐atom‐thick layer of Mo (110). Inset shows corresponding Fourier‐transformed image. Reproduced with permission.^[^ ^43^ ^]^ Copyright 2020, Wiley‐VCH. Panel IV: e‐beam fabrication of monolayer Mo membranes. p) Schematic illustration of fabrication of monolayer Mo membrane from freestanding monolayer MoSe_2_ film. q) STEM‐ADF image of Mo membrane embedded in monolayer MoSe_2_ film. Mo membrane regions are highlighted in false green. r–s) STEM‐ADF images showing r) monolayer MoSe_2_ film and s) monolayer Mo membrane. Atomic models of monolayer MoSe_2_ film and Mo membrane are shown in (t) and (u), respectively. Scale bars: 2 nm in (q) and 0.5 nm in (r) and (s). Reproduced with permission.^[^ ^44^ ^]^ Copyright 2018, Wiley‐VCH.

In another study, atomically thin freestanding Au membranes were fabricated through the local dealloying of Au and Ag atoms using e‐beam irradiation.^[^ ^41^ ^]^ Continuous electron irradiation resulted in the formation of ferromagnetic nanoribbons with widths of 0.65–0.9 nm. **Figure** **6**a shows an illustration of the process used for the fabrication of Au membranes, wherein the e‐beam causes the removal of Ag atoms. The remaining Au atoms form a monolayer with a hexagonal close‐packed lattice. High‐resolution TEM and STEM images of a thus‐formed Au membrane are shown in Figure 6b–c. The lattice parameters (R_2_, R_3_, and R_4_) of the Au membranes were calculated from the high‐resolution TEM images, as shown in Figure 6d, and were found to be 0.267 (±0.004), 0.268 (±0.005), and 0.267 (±0.004) nm, respectively. The line profile of the Au membrane was also determined, as shown in Figure 6e, highlighting the clear differences in the image intensities for the membrane and bulk regions. The number of atomic layers can be seen in the color scale images in Figure 6f–g. It is clear that the central region presents a monolayer Au membrane.

**Figure 7:**
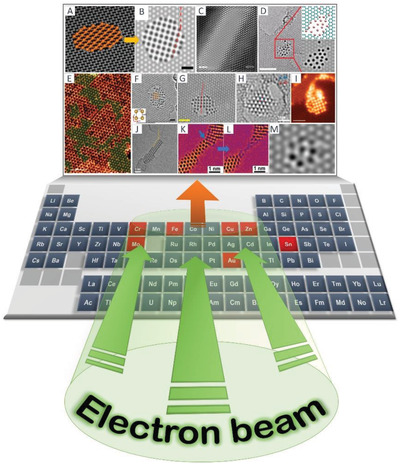
In situ (S)TEM e‐beam‐driven reactions are an ideal approach for forming single‐atom‐thick freestanding 2D metals membranes. A,B,F) Reproduced with permission.^[^ ^19^ ^]^ Copyright 2014, American Association for the Advancement of Science. C) Reproduced with permission.^[^ ^41^ ^]^ Copyright 2019, American Chemical Society. D) Reproduced with permission.^[^ ^37^ ^]^ Copyright 2013, The American Society of Mechanical Engineers. E) Reproduced with permission.^[^ ^44^ ^]^ Copyright 2018, Wiley‐VCH. G) Reproduced with permission.^[^ ^36^ ^]^ Copyright 2020, American Chemical Society. H) Reproduced with permission.^[^ ^24^ ^]^ Copyright 2015, American Chemical Society. I) Reproduced with permission.^[^ ^40^ ^]^ Copyright 2016, IOP Publishing. J) Reproduced with permission.^[^ ^39^ ^]^ Copyright 2020, Wiley‐VCH. K,L) Reproduced with permission.^[^ ^42^ ^]^ Copyright 2020, Wiley‐VCH. M) Reproduced with permission.^[^ ^38^ ^]^ Copyright 2020, Springer Nature.

The thinning of 2D materials can result in the formation of novel materials such as N‐doped graphene and Mo nanoribbons. Mendes et al.^[^ ^42^ ^]^ subjected 2D Mo_2_Ti_2_C_3_ monolayers to e‐beam irradiation in the TEM mode at 300 kV. The freestanding monolayers were irradiated for 6 min, and the thinning process was monitored. EELS showed that the irradiated areas of the Mo_2_Ti_2_C_3_ monolayers remained stable for the first 2 min. However, this was followed by the formation of sites with a high Mo content and regions with a high C content. These sites and regions evolved during the remaining 4 min of irradiation. In the first case, e‐beam irradiation led to the constant removal of Mo atoms until a network of Mo nanoribbons was formed. Continuous irradiation meant that the Mo nanoribbons were thinned continuously until a single‐atom chain formed and ruptured. In the second case, Ti and Mo were removed gradually. The thinning process resulted in the formation of monoatomic N‐doped graphene areas, as shown in Figure 6h–j. These regions have an average size of 3 nm and also contain N. The doping of these graphene regions with N can be attributed to the decomposition of tetrabutylammonium (TBA^+^) during the e‐beam irradiation process. The presence of TBA+, in turn, can be ascribed to the delamination process used for forming the Mo_2_Ti_2_C_3_ monolayers. Figure 6h’–j’ presents a simulated TEM image of the N‐doped graphene regions formed by the thinning of the Mo_2_Ti_2_C_3_ monolayers. Figure 6j–m shows FFT‐filtered snapshots of Mo nanoribbons over time. The yellow arrow indicates the point where Mo ribbon was bound to an amorphous‐like area indicated by the red arrow. Upon exposure to the electron beam, the Mo nanoribbon is thinned forming a single‐atom chain prior to its breakage.

Similarly, Liu et al.^[^ ^73^ ^]^ were able to form Mo_5_S_4_ nanoribbons by thinning MoS_2_ monolayers through e‐beam irradiation. They found that the irradiation of the MoS_2_ monolayers using a TEM system at 80 kV caused the formation of defects, which evolved into holes with a diameter of 3–6 nm. Under continuous irradiation, the connecting regions between the holes narrowed to form nanoribbons with a width of 0.35 nm and bandgap of 0.77 eV. The authors claimed that, given the ease of controlling the formation of these nanoribbons and their high stability, they can be used in various functional devices.

The development of controllable methods for tuning the properties of 2D materials by either reducing their size or creating new nanostructured materials is pivotal to the realization of next‐generation functional devices. Si et al.^[^ ^43^ ^]^ used an aberration‐corrected TEM system operating at voltages higher than 80 kV to preferentially remove C from 2D *α*‐Mo_2_C crystals. The irradiation process led to the formation of thin rectangular Mo structures with lattice parameters of 0.233 and 0.167 nm, as shown in Figure 6n and having the (110) crystal orientation. Simulation images (see Figure 6o) confirmed the formation of a two‐atom‐thick layer. Furthermore, while 2D Mo membranes could be formed under irradiation at 200 and 300 kV, they were not formed when the voltage used was 80 kV. This strongly suggests that it is possible to control the formation of ultrathin 2D Mo membranes with unique properties from 2D *α*‐Mo_2_C crystals by tuning the energy of the incident e‐beam.

2D Mo membranes have also been fabricated by selectively removing Se atoms from monolayer MoSe_2_ using a focused beam in the STEM mode.^[^ ^44^ ^]^ The weak nature of the Mo–Se bond makes the formation of 2D Mo membranes embedded within the MoSe_2_ lattice easy. Figure 6p shows an illustration of the method used to fabricate 2D Mo with a focused e‐beam at 80 kV. The focused e‐beam is scanned across different regions of the MoSe_2_ lattice, leading to the formation of 2D Mo membranes in selected regions of the initial MoSe_2_ crystal, as indicated by the green‐shaded areas in Figure 6q. Figure 6r shows an atomic‐resolution STEM image of a MoS_2_ film with a lattice parameter of 0.33 nm. For comparison, Figure 6s shows a high‐resolution STEM image of a 2D Mo site with a lattice parameter of 0.27 nm. The authors thoroughly characterized the 2D Mo membrane as well as the various types of vacancies formed by the sputtering of S atoms. They also determined that the 2D Mo membranes were stable under electron irradiation and that this top‐down thinning approach, which uses a focused e‐beam, is very efficient and can be extended to fabricate other types of 2D metal membranes as well. Moreover, the use of the appropriate acceleration voltage can aid the formation of novel 2D materials.

## 4 Summary of the Experimental Parameters

There are several important parameters in the fabrication of 2D metal/metallene or 2D metal/ metallene oxides using electron beams as the driving mechanism for their fabrication. Typically these include the electron acceleration voltage, the electron beam dose (current, irradiation time) etc. These key parameters of course vary depending on the specimen composition and they are summarized in the tables presented below. The tables are arranged in two fabrication approaches, namely, growth in supports such as a graphene pore or hole and layer thinning through electron beam irradiation.

In the case of forming 2D membranes (nanoribbon, clusters) in a graphene pore (**Table** **1**), most of the experiments were carried out using a parallel electron beam mode (TEM), with the exception of a 2D CuO membrane being formed. Because graphene pores serve as the lateral support system, these fabrication experiments were performed with low acceleration voltages (i.e., at or <80 kV) and relatively low electron beam doses were implemented. The irradiation time significantly depends on the reactivity of the material to the electron beam. At times, the formation process can be fast (≈3 s in case of an Fe 2D membrane) or relatively slow (≈316 s for a Au 2D nanoribbon). In these instance no additional heating was implemented.

**Table 1:** Summary of the main electron beam parameters for the in‐situ fabrication of freestanding single atom thick 2D metals/metallenes and 2D metals/metallenes oxides suspended in graphene holes

Initial materials	Final materials	Electron beam mode	Acceleration voltage	Electron beam dose/ current	Irradiation time	Heating	Refs.
Fe clusters	2D Fe membrane	TEM	80 kV	0. 625 × 10^6^ ‐ 6.25 × 10^6^ *e*/s nm^2^	≈3 s	No	[19]
Amorphous of C, Cr, O	2D Cr membrane	TEM	80 kV	≈4.06 × 10^6^ *e*/s nm^2^	≈20 s	No	[36]
ZnO nanoparticles	2D ZnO membrane	TEM	80 kV	≈10^7^ *e*/s nm^2^	≈180 s	No	[24]
CuO clusters	2D CuO membrane	STEM	60 kV	12.5 × 10^7^ *e*/s nm^2^ (Probe current)	N/A	No	[40]
Au nanoparticles	2DAu Nanoribbon	TEM	80 kV	≈2.38 × 10^6^ *e*/s nm^2^	≈316 s	No	[39]
Pt nanoparticles	2D Pt cluster	TEM	60 kV	≈10^7^ *e*/s nm^2^	N/A	No	[37]
Sn Acetylacetonate	2D Sn cluster	TEM	80 kV	≈2.38 × 10^6^ *e*/s nm^2^	N/A	No	[38]

The main parameters governing the thinning of 2D nanomaterials to form freestanding single atom thick 2D metals/metallenes and 2D metals/metallenes oxides presented in this review using an electron beam are provided in **Table** **2**. The most relevant parameters when thinning multi‐layer materials or binary alloys in TEM and STEM modes are the acceleration voltage in combination with the irradiation time and total dose that the material is exposed to. Again, as mentioned above, these parameters are intrinsically connected with the nature of the material studied. When localized thinning and etching using electron beams are investigated (e.g., to create point defects or pores) using a condensed beam (STEM) appears to be the most successful technique. It is also important to mention that in‐situ heating is also often used to help thin 2D materials both alone and in combination with electron beam to enhance the desired thinning process.

**Table 2:** Summary of main electron beam parameters and techniques for thinning 2D nanomaterials for the formation of freestanding single atom thick 2D metals/metallenes and 2D metals/metallenes oxides

Initial materials	Final materials	Electron beam mode	Acceleration voltage	Electron beam dose/ current	Irradiation time	Heating	Refs.
Au_25_Ag_75_ alloy	Au membranes and nanoribbons	TEM	200, 300 kV	3.125 × 10^6^ ‐ 25 × 10^6^ *e*/s nm²	0 ‐ 24 s	No	[41]
Mo_2_Ti_2_C_3_	N‐doped graphene, Mo nanoribbons	TEM	80, 300 kV	N/A	360 s	No	[42]
*α*‐MoC_2_	2D Mo (@ 200 and 300 kV)	TEM	80, 200, 300 kV	N/A	360 s	No	[43]
Black phosphorus	Layer thinning with protective graphene layer	TEM	80 kV	1.5 × 10^7^ ‐ 2.5 × 10^8^ *e*/s nm²	Based on deposited dose	No	[68]
SnS_2_ and SnSe_2_	Layer thinning	TEM	80, 200, 300 kV	12.5 × 10^7^ *e*/s nm^2^	N/A	20–400 °C	[69]
WS_2_	Layer thinning	TEM/STEM	200 kV	N/A	360 s	800 °C	[67]
MoS_2_	MoS_2_ nanoribbons	TEM/STEM	60 kV	27.5 × 10^7^ *e*/s nm^2^ (probe current)	N/A	800 °C	[70]
MoS_2_	Multiple new MoS_2_ structures and phases	TEM/STEM	200 kV	56.25 × 10^7^ *e*/s nm^2^ (probe current)	N/A	850 °C	[71]
MoSe_2_	2D Mo membrane	STEM	80 kV	1 × 10^6^ ‐ 7.4 × 10^7^ *e*/s nm² (probe current)	5–140 s	No	[44]
Monolayer graphene	Graphene etching forming nanoribbons	STEM	100, 60 kV	10.6 × 10^7^ *e*/s nm² (probe current)	N/A	No	[66]
MoS_2_ / WS_2_ vertical heterostructure	Controlled formation of pores	STEM	80 kV	4 × 10^4^ ‐ 2 × 10^8^ *e*/s nm²	10 and 20 s	No	[72]

**Table** **3** also summarizes other techniques apart from electron beam that have been utilized to thin 2D nanomaterials. There are many parameters involved in the thinning process and each technique pursues its own control parameters, which are also material dependent. Unlike the S/TEM experiments, these other techniques require that the 2D nanomaterial is supported (typically on Si/SiO_2_), making it harder and in many cases preventing a direct real‐time observation of the thinning process.

**Table 3:** Summary of alternate techniques for thinning 2D nanomaterials

Technique	Initial material	Final material	Main controlling parameter	Refs.
Ion irradiation	Graphene and MoS_2_ on Si/SiO_2_ substrate	Track defect formation in the lattice	He^+^, Ar^+^ and Ne^+^ ion beams Energy: 1 ‐ 30 kV	[55]
Ion irradiation	Multi‐layer MoS_2_ on Si/SiO_2_ substrate	Layer thinning	Ga^+^ ion beam Energy: 10 kV	[56]
Ion irradiation	Multi‐layer Black phosphorus on Si/SiO_2_ substrate	Layer thinning	Ar^+^ ion beam Energy: 5 ‐ 80 eV	[57]
Laser irradiation	Multi‐layer MoS_2_ on Si/SiO_2_ substrate	Layer thinning	532 nm laser Power: 1.5 mW Time: 0.2 s scan^−1^	[58]
Plasma treatment	Multi‐layer MoS_2_ on Si/SiO_2_ substrate	Layer thinning	Ar^+^ plasma Power: 50 W Pressure: 40 Pa Time: 0 ‐ 115 s	[59]
Plasma treatment	Multi‐layer MoSe_2_ on Si/SiO_2_ substrate	Layer thinning	SF6 (4.5 sccm) + N2 plasma (1.0 sccm) Power density: 0.8 and 1.2 mW cm^−^³ Time: 80 and 40 s steps	[60]
SEM (ice lithography)	Monolayer MoS_2_ on Si/SiO_2_ substrate	Patterning by etching of monolayer	Electron beam Amorphous ice deposition at 130 K Energy: 10 kV Pressure: 10^‐6^ mbar	[61]
Chemical treatment	Monolayer hexagonal boron nitride on Si/SiO_2_ substrate	Patterning by etching of monolayer	Electron beam Water vapor as etch precursor Energy: 25 and 15 kV Time: 0–500 s Temperature range: 25–325 °C	[62]
Thermal treatment	Multi‐layer Black phosphorus on Si/SiO_2_ substrate	Layer thinning	Annealing in flow of air and N_2_/H_2_ mixture Temperature: 330–360 °C Time: 120 s	[63]
Thermal treatment	Multilayer Bi_2_Se_3_ and Sb_2_Te_3_	Layer thinning	Annealing Temperature: 510 °C for Bi_2_Se_3_, 490 °C for Sb_2_Te_3_ Time: 10 min	[64]

## 5 Outlook

Although the list of predicted 2D materials^[^ ^74^ ^]^ has been increasing steadily in the last few years, it remains a significant challenge to even observe these materials experimentally. For example, only a limited number of freestanding 2D metals/metallenes have been synthesized so far (**Figure** **7**). In addition, only a few elements have been synthesized in the form of freestanding structures, and the periodic table remains to be fully explored with the expectation that even more novel materials that exhibit unique properties will be discovered. Various types of binary 2D structures can be formed at the small scale (e.g., nanoribbons). These materials can, in turn, serve as templates for the fabrication of novel 2D materials (e.g., 2D metals/metallenes).

TEM and STEM have immense potential in the controlled fabrication of novel 2D materials that can be incorporated in next‐generation devices that can revolutionize the current state of technology. For example, they can lead to the development of more efficient electronic devices and improve the energy storage capability of existing ones. Both techniques can be used for the controllable engineering of 2D materials, which can be accomplished through both bottom‐up and top‐down approaches. In both cases, it is possible to form new materials. We would also like to highlight our vision of electron engineering as a mass production method that can be realized through the development of next‐generation multi‐beam systems, automation and artificial intelligence techniques. We have described this view in greater detail in a recently published report.^[^ ^30^ ^]^

It is also important to point out that the usage of electron beams goes beyond the investigation and manipulation of 2D materials and it reaches applications in a number of fields. Apart from being a standard method used in the characterization of nanomaterials with high spatial and temporal resolution, electrons are also used to obtain spectroscopic information about specimens,^[^ ^75^, ^76^ ^]^ as well as trigger catalytic processes.^[^ ^77^ ^]^ Electron beams also find application in the use of additive manufacturing, also known as electron beam melting or 3D printing with electrons.^[^ ^78^, ^79^, ^80^ ^]^ The fabrication of precise nanoparticle arrays and electric circuits are also possible using electron lithography.^[^ ^81^, ^82^, ^83^, ^84^ ^]^ The manipulation of polymers has also been accomplished with electron beams and finds most of its applications to crosslink polymers.^[^ ^85^, ^86^, ^87^ ^]^

## 6 Conclusion

Novel 2D metals and metal oxides show unique properties and hence have significant potential for use in a range of applications, making them both interesting and challenging research subjects. Several approaches have been reported for synthesizing these materials and confirming their existence. However, given the recent advancements in reducing chromatic and spherical aberrations, which allow for the use of low acceleration voltages while maintaining a high atomic resolution—this is particularly important with respect to low‐dimensional materials—HRTEM has emerged as an ideal approach for studying 2D metals/metallenes and 2D metals/metallene oxides and confirming their existence. During HRTEM, the irradiation of the e‐beam leads to in‐situ reactions involving the test specimen, resulting in novel 2D materials. In this review, we summarized the recent achievements made with regard to the use of in situ (S)TEM for studying the freestanding configurations of 2D metals/metal oxides, which has remained challenging till now. With respect to in situ (S)TEM, there are two primary approaches for forming freestanding monoatomic membranes. The first is using graphene pores as templates, so that the e‐beam causes the metal/metal oxide atoms to fill the graphene pores and crystallize and thus form a freestanding 2D crystal membrane. The second is e‐beam sputtering or the selective etching of metal alloys (e.g., Au–Ag alloys) or thick complex initial materials (e.g., Mo_2_Ti_2_C_3_, MoSe_2_, Mo_2_C, or MoS_2_) to obtain freestanding single‐atom‐thick 2D metal membranes. The data show a growing number of 2D metals/metallenes and 2D metals/metallene oxides having been confirmed and point to a bright future for further discoveries of these 2D materials.

## Conflict of Interest

The authors declare no conflict of interest.
